# Toward unidirectional switches: 2-(2-Hydroxyphenyl)pyridine and 2-(2-methoxyphenyl)pyridine derivatives as pH-triggered pivots

**DOI:** 10.3762/bjoc.8.110

**Published:** 2012-06-29

**Authors:** Christina Tepper, Gebhard Haberhauer

**Affiliations:** 1 Institut für Organische Chemie, Fakultät für Chemie, Universität Duisburg-Essen, Universitätsstraße 7, D-45117 Essen, Germany

**Keywords:** CD spectroscopy, chirality, molecular modeling, molecular switches, unidirectional movements

## Abstract

The pH-induced switching process of 2-(2-hydroxyphenyl)pyridine and 2-(2-methoxyphenyl)pyridine derivatives was investigated with the help of UV spectroscopy. Quantum chemical calculations at the B3LYP/6-31G* level of theory were performed to show that in the case of 2-(2-methoxyphenyl)-3-methylpyridine and 2-(2-hydroxyphenyl)-3-methylpyridine the rotation during the switching process proceeds unidirectionally at the molecular level. If a 2-(2-methoxyphenyl)pyridine derivative is fixed to a chiral cyclopeptidic scaffold, a unidirectional progress of the rotation is achieved macroscopically.

## Introduction

**Scheme 1:**
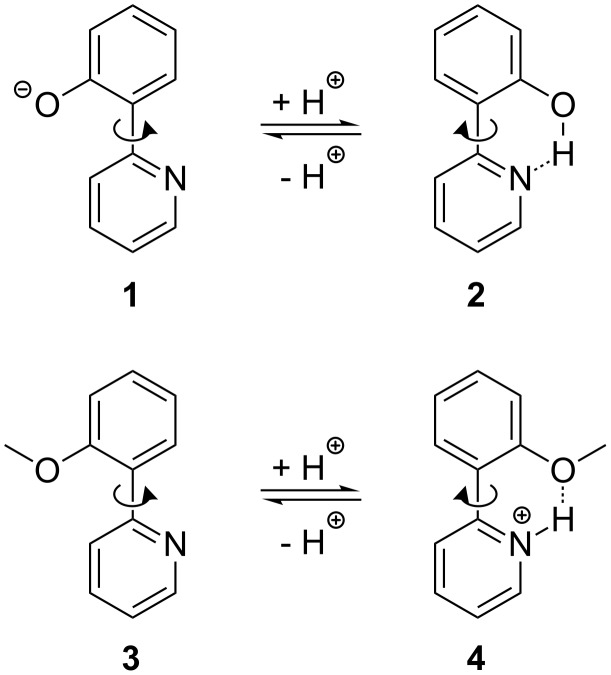
Principle of the switching mechanism of 2-(2-hydroxyphenyl)pyridine (**2**) and 2-(2-methoxyphenyl)pyridine (**3**).

A current topic that is rapidly expanding and the subject of extensive research is the design of molecular analogues of mechanical devices that are able to carry out movements powered by external stimuli [1–14]. Numerous examples of switches [15–18], rotors [19–22] and shuttles [23–28] that can be controlled chemically, electrochemically, thermally or by illumination have been described. One of the most challenging aspects in the design of molecular devices is the creation of synthetic molecular motors, which utilize the unidirectional movements of smaller parts thereof and which, thus, should be able to perform a physical task [2]. One important requirement for the construction of a molecular motor is that at least one movement of the motor proceeds unidirectionally. Such a unidirectional movement was already realized for rotations around N–N [29] and C–C [19–20,22,30–31] double bonds, around C–C single bonds [21,32–36], mechanical bonds [37–38] and in metal complexes [39–48]. A system for which unidirectional movement was realized, by making use of two different concepts, is the 2,2′-bipyridine unit [32,34–35]. Rotation around the C–C bond that connects the two pyridine units is induced by the addition of a metal salt, which leads to the corresponding metal complexes. The back rotation is caused by the removal of the metal ion by the addition of cyclam, which complexes the metal ions better than the 2,2′-biypridine unit does. 2-(2-Hydroxyphenyl)pyridine and 2-(2-methoxyphenyl)pyridine should show a similar behavior (see Scheme 1) and therefore should also be applicable as pivots in molecular switches. While 2,2′-biypridine derivatives have been intensively used for molecular devices [49–56], 2-(2-hydroxyphenyl)pyridine and 2-(2-methoxyphenyl)pyridine derivatives, to our best knowledge, have only been used as ligands for metal complexes, but not for the construction of molecular switches [57–63]. Similar compounds, such the bipyridindiols, were studied as molecular half-subtractors, but they did not work unidirectionally [59]. Here we investigate their usability as pivots, especially for unidirectional rotations.

## Results and Discussions

### Concept

The switching process for 2-(2-hydroxyphenyl)pyridine (**2**) and 2-(2-methoxyphenyl)pyridine (**3**) should be achieved by the addition of acid and base, respectively. In the case of **2** the dihedral angle θ_N–C–C–C(O)_, which describes the relative orientation of the two aromatic units to one another, amounts to 0° [62–63]. This conformation is stabilized by the internal hydrogen bridge between the hydroxy group of the phenol and the nitrogen of the pyridine. A deprotonation of **2** to the penolate **1** leads to a rotation around the C–C bond to the most stable conformation of **1**, which has a dihedral angle θ_N–C–C–C(O)_ of 180°. This conformation allows a maximum conjugation over both aromatic rings and avoids the repulsion between the nitrogen lone pairs and the negatively charged oxygen atom. A similar behavior should be valid for 2-(2-methoxyphenyl)pyridine (**3**): in a neutral or basic environment the conformation showing a dihedral angle θ_N–C–C–C(O)_ of 180° and thus avoiding the repulsive interaction between the free electron pairs of the nitrogen and the oxygen atoms, should be the more stable one (Scheme 1). A protonation of the nitrogen inverts the relative energies of the two conformers. Due to the internal hydrogen bridge the conformer of **4** having a dihedral angle of 0° is the more stable one.

**Figure 1:**
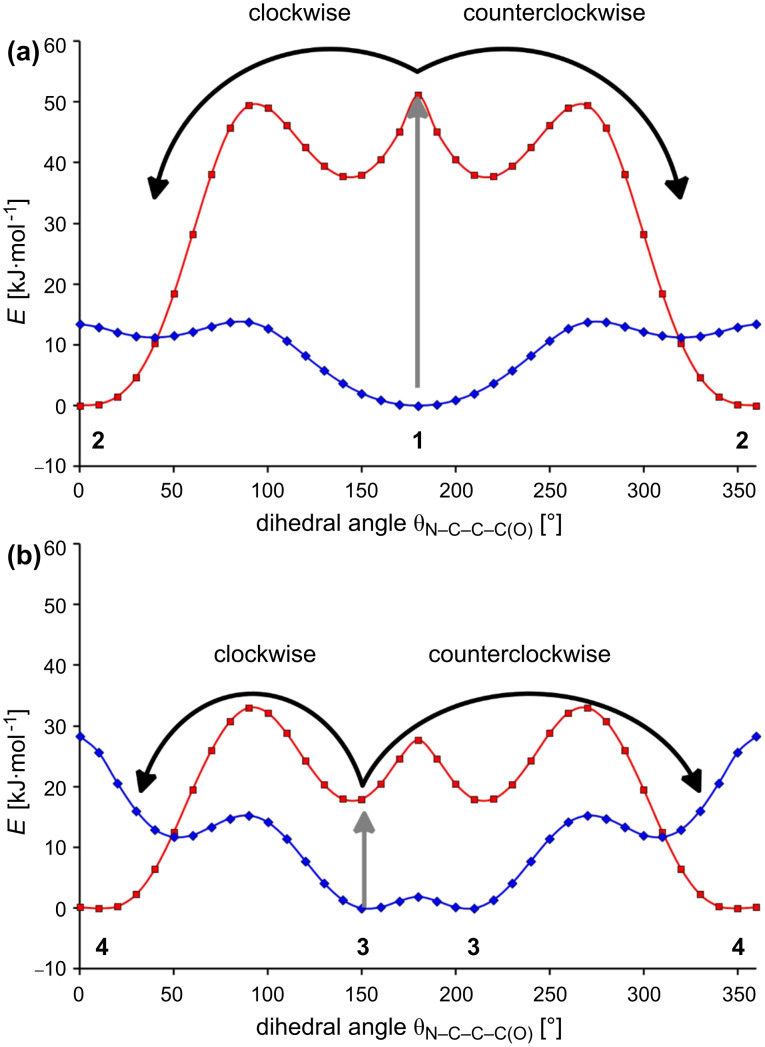
(a) Calculated energy profiles of the pyridine derivatives **1** (blue) and **2** (red) in relation to the dihedral angles θ_N–C–C–C(O)_ by use of B3LYP/6-31G*. (b) Calculated energy profiles of the pyridine derivatives **3** (blue) and **4** (red) in relation to the dihedral angles θ_N–C–C–C(O)_ by use of B3LYP/6-31G*.

In order to verify these assumptions, the rotational barriers for **1**–**4** were calculated by using B3LYP and the 6-31G* basis set (Figure 1) [64]. Indeed, for **2** and **4** (red curves), which are able to form internal hydrogen bonds, the conformations with a dihedral angle of 0° are the more stable ones. In the case of **1** and **3** (blue curves), in which the repulsive interaction between the lone pairs is the dominant one, the conformations exhibiting a dihedral angle of about 180° are energetically preferred. A closer look at Figure 1 shows, however, that both systems as such are not suitable for use as a unidirectional pivot. If the phenolate **1** is protonated (grey arrow in Figure 1a), the most-stable conformation of the phenol **2** can be reached either by a clockwise or a counterclockwise rotation around the C–C single bond (black arrows in Figure 1a). As the transition states for both rotations show equal energies, both processes are equiprobable. The same is true for the protonation of the methoxyphenylpyridine **3** (black arrows in Figure 1b). Thus, protonation of **1** and **3** does not lead to unidirectional rotation at the molecular level.

**Scheme 2:**
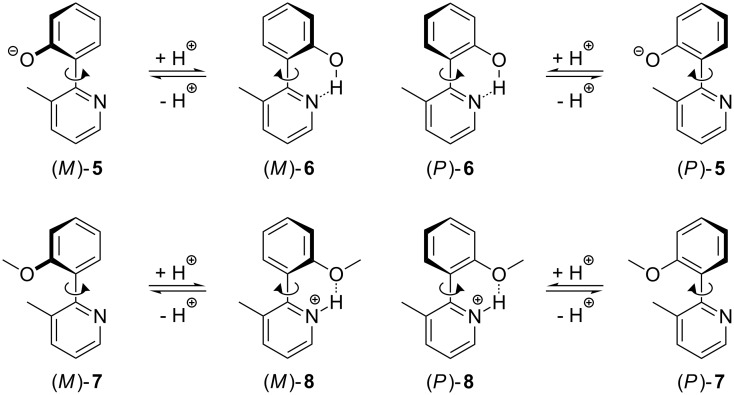
Principle of the switching mechanism of 2-(2-hydroxyphenyl)-3-methylpyridine (**6**) and 2-(2-methoxyphenyl)-3-methylpyridine (**7**).

**Figure 2:**
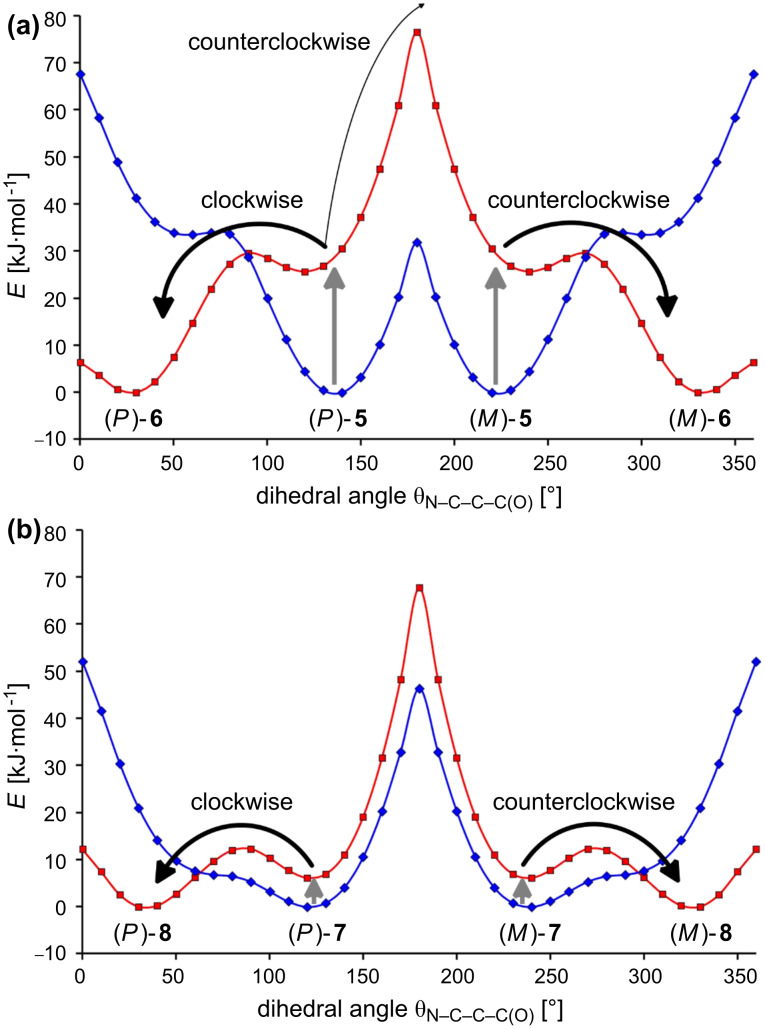
(a) Calculated energy profiles of the 3-methylpyridine derivatives **5** (blue) and **6** (red) in relation to the dihedral angles θ_N–C–C–C(O)_ by use of B3LYP/6-31G*. (b) Calculated energy profiles of the 3-methylpyridine derivatives **7** (blue) and **8** (red) in relation to the dihedral angles θ_N–C–C–C(O)_ by use of B3LYP/6-31G*.

In order to obtain a switching unit that shows a unidirectional rotation process caused by protonation, the methyl derivatives of **1**–**4**, i.e., **5**–**8** (Scheme 2) were investigated by using quantum chemical calculations at the B3LYP/6-31G* level of theory. Due to the methyl group, all conformations with a dihedral angle θ_N–C–C–C(O)_ of 180° represent energy maxima, and the minima at dihedral angles of about 130° are pairwise enantiomeric and separated by high energy barriers (Figure 2). Now the rotations around the C–C bonds caused by the protonation of the species **5** and **7** (gray arrows in Figure 2) take place in a unidirectional manner at the molecular level: If for example (*P*)-**5** is protonated, a clockwise rotation to the most stable conformation of **6** is the energetically preferred process (thick black arrow in Figure 2a). The energy barrier for this movement amounts to only a few kJ·mol^–1^. In contrast, for a counterclockwise rotation, a transition state must be overcome that is more than 45 kJ·mol^–1^ higher in energy (thin black arrow in Figure 2a). Thus, the rotation triggered by the protonation of (*P*)-**5** proceeds unambiguously clockwise. On the other side, a protonation of (*M*)-**5** leads to a unidirectional counterclockwise movement. Even more pronounced is the unidirectionality for the rotation triggered by the protonation of the 3-methylpyridine **7**: If (*P*)-**7** is protonated, the difference between the energy of the rotation barriers for the clockwise and the counterclockwise rotation around the C–C bond between the aromatic units, amounts to more than 55 kJ·mol^–1^. According to the Boltzmann distribution between the two transition states, the rotation is almost completely (>99.9999%) unidirectional at 298 K.

It must be emphasized that the rotation caused by the protonation of **5** and **7** is unidirectional only with respect to a single molecule. In equilibrium, the ratio between the *P* and *M* enantiomers amounts to 1:1. As all *P* enantiomers perform a clockwise rotation and all *M* enantiomers a counterclockwise rotation, the whole process (sum of all single processes) is not unidirectional. One possibility to make the whole process at least partly unidirectional is to transform the enantiomeric conformers with a dihedral angle of about 150° into diastereomers that are different in energy. Due to the energetic gap between the diastereomers there will be an excess of one in solution. As the majority of the switches will now rotate in one direction (e.g., clockwise) and the minority will rotate in the other direction (e.g., counterclockwise), the whole process will now exhibit net unidirectionally (e.g., clockwise).

### Proof of the switching process

**Figure 3:**
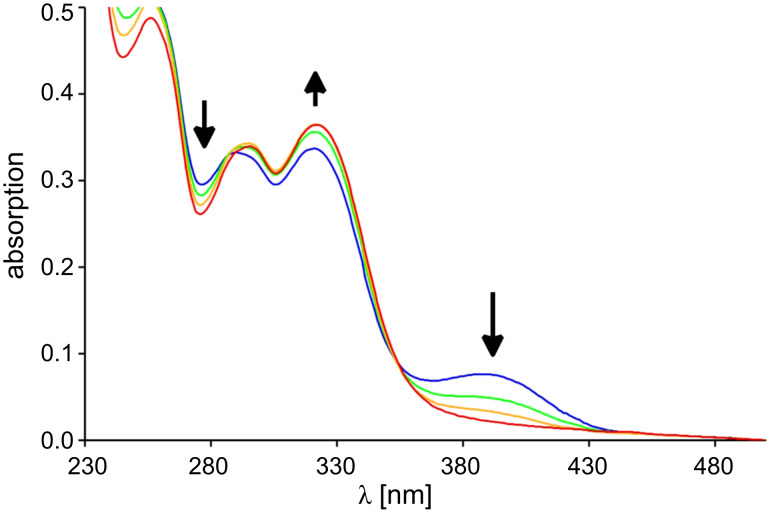
UV spectral change of phenolate **5** (blue) in dichloromethane (c = 5.6 × 10^−5^ M) at 20 °C upon addition of 10 (green), 20 (yellow) and 60 equiv TFA (**6**, red).

**Figure 4:**
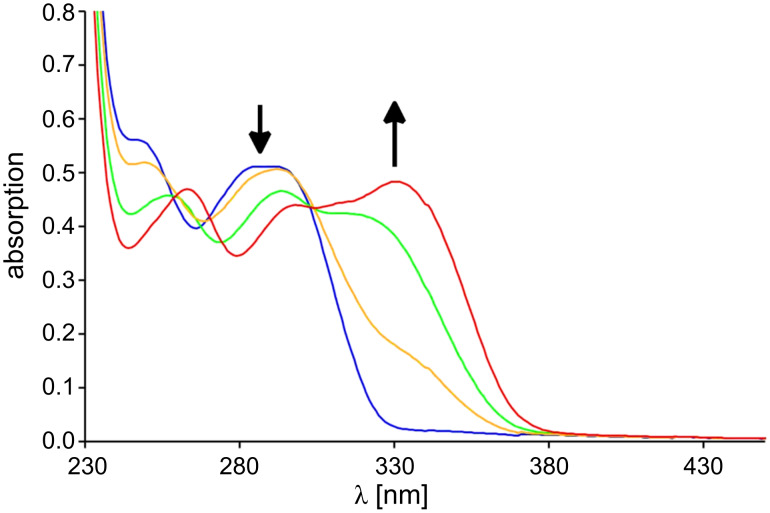
UV spectral change of 3-methylpyridine **7** (blue) in dichloromethane (c = 5.6 × 10^–5^ M) at 20 °C upon addition of 1 (yellow), 2.5 (green) and 20 equiv TFA (**8**, red).

The switching process can easily be observed by UV spectroscopy. The protonation was accomplished by the addition of several equivalents of trifluoroacetic acid (TFA). If TFA is added to the phenolate **5** (which was previously prepared by the addition of 50 equiv tetrabutylammonium fluoride to **6**) the absorption band at 388 nm disappears and simultaneously the band at 322 nm increases, while the band at 276 nm decreases (Figure 3). The thus obtained spectrum resembles the spectrum of the phenol **6**. Thus the deprotonation–protonation process is reversible. If TFA is added to a solution of **7** in dichloromethane (DCM), a new absorption band at 331 nm appears and the peak at 290 nm decreases (Figure 4). This is nothing else but a bathochromic shift of the band at 290 nm of **7** to higher wavelengths. The addition of tetrabutylammonium fluoride leads to the original spectrum.

**Figure 5:**
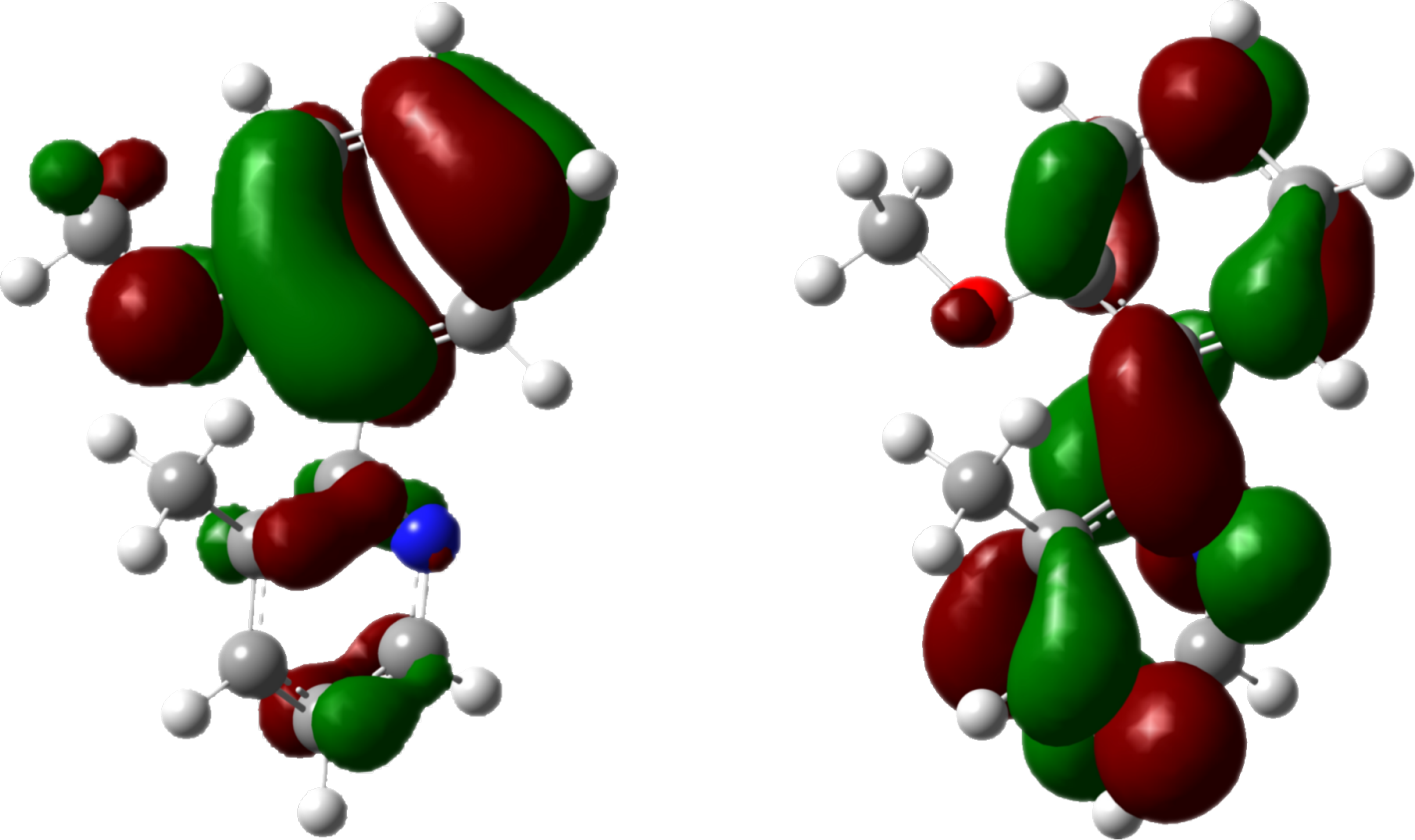
HOMO (left) and LUMO (right) of the 3-methylpyridine **7** calculated by using B3LYP/6-31G*.

The bathochromic shift during the transition from 3-methylpyridine **7** to the protonated species **8** can be explained on the basis of time-dependent density functional theory (TD-DFT) with the B3LYP functional and by employing the 6-31G* basis set. The absorption band at 290 nm for the 3-methylpyridine **7** as well as the absorption band at 331 nm for the protonated 3-methylpyridine **8** are dominated by the transition of an electron from the HOMO to the LUMO (π→π* transition; Figure 5). In both cases the HOMO is represented mainly by the p_π_ orbitals of the methoxyphenyl unit, whereas in the LUMO the p_π_ orbitals of the pyridine system prevail. A protonation of the nitrogen atom leads to an energetic decrease of all orbitals. As the HOMO exhibits only a small coefficient at the protonated nitrogen atom, the energetic lowering of the HOMO (Δε = −3.89 eV) caused by the protonation is less pronounced than the one observed for the LUMO (Δε = −5.32 eV). The resulting decrease of the HOMO–LUMO gap is responsible for the bathochromic shift of the energetically lowest π→π* band. A similar explanation can be found for the hypsochromic shift caused by the protonation of the phenolate anion **5**. In summary, both the phenolate **5** and the pyridine **7** can be used as pH-triggered pivots, and according to calculated energy profiles the rotational movements during the switching process proceed in both cases unidirectionally at the molecular level.

### Unidirectionality of the switching process

**Scheme 3:**
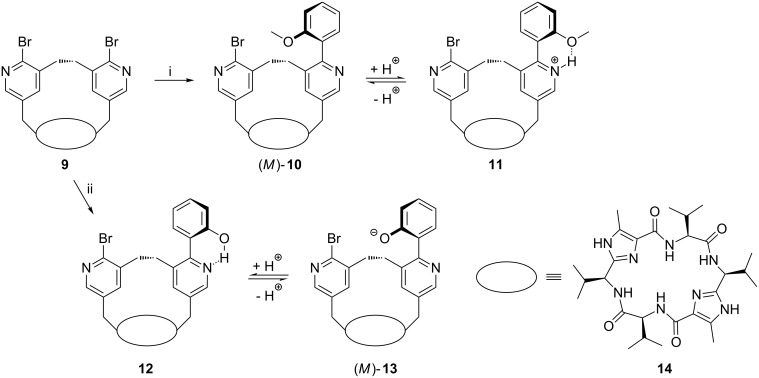
Synthesis of the methoxyphenylpyridine switch **10** and the hydroxypyridine switch **12**; reaction conditions: (i) 2-methoxyphenylboronic acid, Pd(PPh_3_)_4_, K_2_CO_3_, H_2_O/dioxane, 80 °C, 16%; (ii) 2-hydroxyphenylboronic acid, Pd(PPh_3_)_4_, K_2_CO_3_, H_2_O/dioxane, 80 °C, 29%.

As already mention above, the whole switching process (sum of all molecular processes) becomes unidirectional if the enantiomeric conformers with a dihedral angle of about 150° are transformed into diastereomers that are different in energy. In order to receive diastereomeric derivatives of 2-(2-hydroxyphenyl)-3-methylpyridine (**6**) and 2-(2-methoxyphenyl)-3-methylpyridine (**7**) we intended to insert these units into a chiral macrocycle. Therefore, we used the macrobicyclus **9** in which a bridge consisting of two pyridine units spans over the peptidic clamp **14** (Scheme 3). Due to the chirality of the clamp, the bridge adopts a specific conformation (in this case the *P* conformation). The desired pyridine switches **10** and **12** can easily be synthesized by a Suzuki reaction of the bipyridine **9** with 2-methoxyphenylboronic acid or 2-hydroxyphenylboronic acid, respectively, using tetrakis(triphenylphosphine)palladium(0) and potassium carbonate as a base in dioxane. In the resulting switches **10** and **13** the more stable diastereomeric conformations should be those in which the methoxy group or the negatively charged oxygen atom, respectively, is turned away from the ethylene unit bridging the two pyridine units. In our case that would be the isomers (*M*)-**10** and (*M*)-**13**.

According to full geometry optimization studies in which calculations at the B3LYP/6-31G* level of theory were performed, the *M* isomer of **13** is more stable than the *P* isomer by 4.4 kJ·mol^−1^. This means that in equilibrium the ratio between the diastereomers amounts to 85:15 in favor of the *M* isomer. In the case of a protonation of the pyridine switch **13** the rotation during the switching process will be in sum unidirectional (in our case counterclockwise). In the case of the methoxyphenylpyridine switch **10** the difference between the *M* and the *P* isomer was calculated with B3LYP/6-31G* to be 2.1 kJ·mol^−1^ in favor of the *M* isomer. According to this calculation the ratio between the diastereomers amounts to 70:30. Unfortunately, there is no hint toward a preference for the *M* conformer of **13** in solution: For example, in the ^1^H NMR spectrum of **13** signals for only one conformer were found, even at lower temperatures, which means that the diastereomers are rapidly interconverting and the ratio between them cannot be determined by ^1^H NMR spectroscopy. In the ^1^H NMR of **10**, the signals of two different conformers are present and the ratio between the conformers is about 60:40. However, it is not possible to determine which isomer is the predominant one from 2D NOESY experiments.

**Figure 6:**
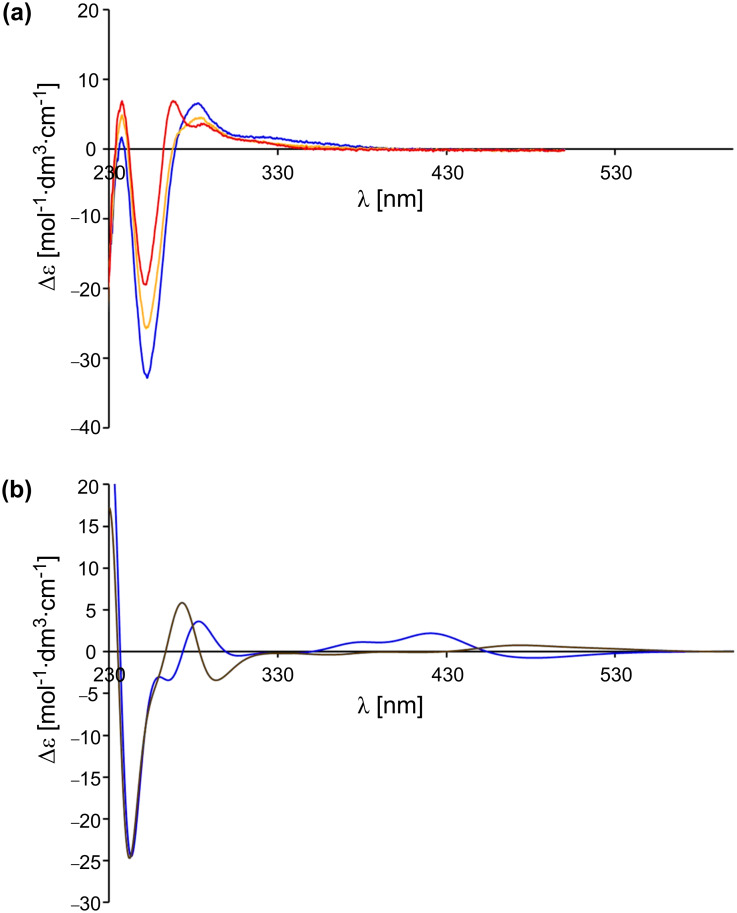
(a) CD spectral change of pyridine switch **13** (blue) in dichloromethane (c = 5.6 × 10^–5^ M) at 20 °C upon addition of 10 (yellow) and 30 equiv TFA (**12**, red). (b) Calculated spectra of (*M*)-**13** (blue) and (*P*)-**13** (brown) by using TD-DFT-B3LYP/6-31G*.

Another possibility to test whether the switches adopt a preferred conformation in solution is through the use of CD spectroscopy. For this purpose, the CD spectrum of **13** in dichloromethane as the solvent was recorded (Figure 6a). Additionally, the CD spectra of (*M*)-**13** and (*P*)-**13** were simulated with the time-dependent density functional theory (TD-DFT) with B3LYP as a functional and by employing the 6-31G* basis set (Figure 6b). TD-DFT calculations were performed at the optimized ground-state geometries of (*M*)-**13** and (*P*)-**13**, calculating the energy, oscillator strength and rotatory strength for each of the 200 lowest singlet excitations. The CD spectra were simulated by overlapping Gaussian functions for each transition, for which the width of the band at 1/*e* height was fixed at 0.3 eV, and the resulting intensity of the combined spectrum was scaled to the experimental values. A comparison between the measured spectrum and the calculated spectra shows that apparently neither the *M* nor the *P* isomer of **13** is predominant in solution. Thus, the rotation, which is caused by the protonation of **13** and which can be observed by a hypsochromic shift of the energetic lowest π→π* band in the UV and CD spectrum, does not proceed with net unidirectionally.

**Figure 7:**
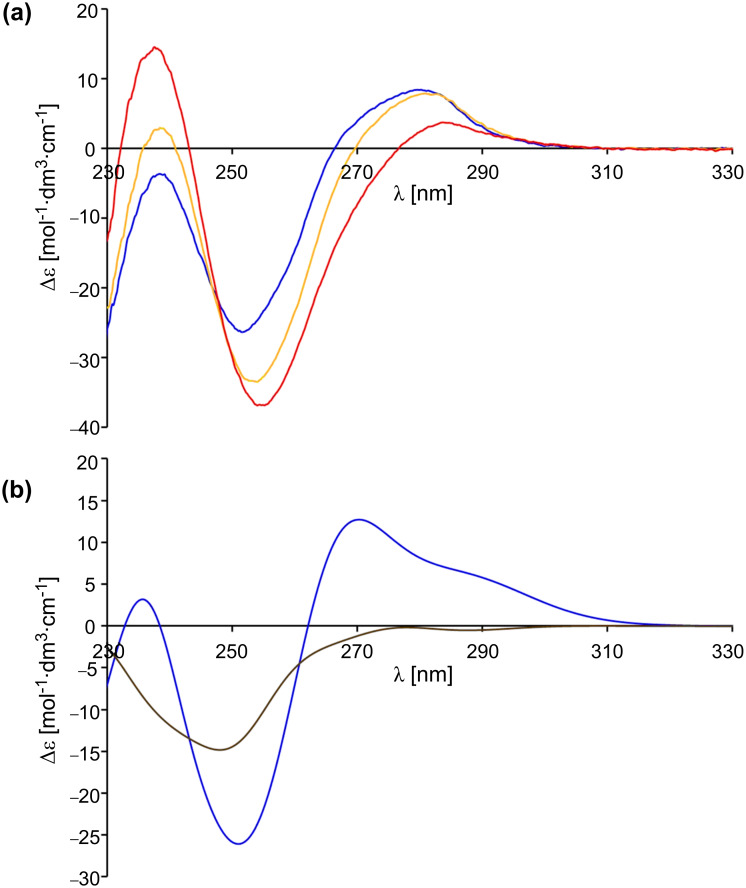
(a) CD spectral change of pyridine switch **10** (blue) in dichloromethane (c = 5.6 × 10^–5^ M) at 20 °C upon addition of 1 (yellow) and 20 equiv TFA (**11**, red). (b) Calculated spectra of (*M*)-**10** (blue) and (*P*)-**10** (brown) by using TD-DFT-B3LYP/6-31G*.

Another result is found for the switch **10**: If we compare the simulated spectra of (*M*)-**10** and (*P*)-**10**, using time-dependent density functional theory (TD-DFT-B3LYP/6-31G*; Figure 7b) with the measured spectrum of **10** in DCM (Figure 7a), it becomes evident that the *M* isomer is the prevailing one. The protonation of the methoxyphenylpyridine switch **10** with TFA leads to a bathochromic shift of the energetically lowest π→π* band in the UV and the CD spectrum. As the *M* isomer is present in solution in excess in relation to the *P* isomer, the whole process is unidirectional even if the extent (percentage) of unidirectionality is small.

## Conclusion

In sum we were able to show that 2-(2-methoxyphenyl)pyridine and 2-(2-hydroxyphenyl)pyridine derivatives can successfully be used as pivots. According to the calculated energy profiles, the rotation movements during the switching of the corresponding 3-methyl derivatives proceed unidirectionally at the molecular level. In the case of 2-(2-methoxyphenyl)pyridine we were able to construct a chiral switch that shows, in relation to the entire ensemble of the molecules, a unidirectional rotation, although the extent of unidirectionality is small.

## Experimental

**General remarks:** All chemicals were reagent grade and were used as purchased. Reactions were monitored by TLC analysis with silica gel 60 F_254_ thin-layer plates. Flash chromatography was carried out on silica gel 60 (230–400 mesh). ^1^H and ^13^C NMR spectra were measured with Bruker Avance DMX 300 and Avance DRX 500 spectrometers. All chemical shifts (δ) are given in ppm. The spectra were referenced to deuterated solvents indicated in brackets in the analytical data. HRMS spectra were recorded with a Bruker BioTOF III instrument. UV absorption spectra were obtained with Jasco J-815 and V-550 spectrophotometers. CD absorption spectra were recorded with a Jasco J-815 spectrophotometer.

**2-(2-Methoxyphenyl)-3-methylpyridine (7):** To a solution of 2-bromo-3-methylpyridine (184 mg, 1.07 mmol), 2-methoxyphenylboronic acid (151 mg, 0.99 mmol) and tetrakis(triphenylphosphine)palladium(0) (45 mg, 3.9 mol %) in dioxane (10 mL), a saturated potassium carbonate solution (1 mL) was added. The mixture was purged with argon for 10 min and afterwards heated to 88 °C for 20 h. After cooling to room temperature, water and ethyl acetate were added. The aqueous phase was extracted with ethyl acetate. The organic layers were combined and dried over magnesium sulfate, and the solvent was removed in vacuo. The residue was purified by column chromatography over silica gel (*n*-hexane/AcOEt 1:1). The product was obtained as a colorless solid (77 mg, 39%). ^1^H NMR (500 MHz, CDCl_3_) δ 7.74 (dd, ^3^*J*_H,H_ = 7.6 Hz, ^4^*J*_H,H_ = 1.9 Hz, 1H, *H*_ar_), 7.59–7.58 (m, 2H, *H*_ar_), 7.37–7.34 (m, 1H, *H*_ar_), 7.09–7.06 (m, 2H, *H*_ar_), 6.99 (d, ^3^*J*_H,H_ = 8.2 Hz, 1H, *H*_ar_), 3.84 (s, 3H, OC*H*_3_), 2.63 (s, 3H, PhC*H*_3_) ppm; ^13^C NMR (125 MHz, CDCl_3_) δ 157.9, 156.9, 155.4, 135.7, 131.1, 129.6, 129.5, 122.0, 121.2, 121.0, 111.3, 55.6, 24.7 ppm; IR (ATR) 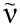: 3057, 2938, 2835, 1574, 1492, 1462, 1292, 1269, 1242, 1161, 1123, 1093, 1024, 870, 800, 750 cm^−1^; UV (CH_2_Cl_2_) λ_max_, nm (log ε): 290 (2.97), 285 (2.97), 249 (3.01); HRMS–ESI (*m*/*z*): [M + H]^+^ calcd for C_13_H_13_NO, 200.1070; found, 200.1116; [M + Na]^+^ calcd for C_13_H_12_NONa, 222.0889; found, 222.0935.

**2-(2-Hydroxyphenyl)-3-methylpyridine (6):** 2-(2-methoxyphenyl)-3-methylpyridine (**7**) (36 mg, 0.18 mmol) was dissolved in dichloromethane (2 mL) and cooled to −70 °C. Then a 1 M boron tribromide solution in dichloromethane (0.6 mL, 0.60 mmol) was added. The mixture was warmed to room temperature overnight. Subsequently the solution was poured into a dichloromethane/water mixture. The organic layer was separated and dried over magnesium sulfate, and the solvent was removed in vacuo. The product was obtained as a colorless solid (32 mg, 95%). ^1^H NMR (500 MHz, CDCl_3_) δ 14.72 (bs, 1H, O*H*), 7.86 (dd, ^3^*J*_H,H_ = 8.0 Hz, ^4^*J*_H,H_ = 1.6 Hz, 1H, *H*_ar_), 7.74–7.73 (m, 2H, *H*_ar_), 7.32–7.28 (m, 1H, *H*_ar_), 7.11–7.09 (m, 1H, *H*_ar_), 7.34 (dd, ^3^*J*_H,H_ = 8.2 Hz, ^4^*J*_H,H_ = 1.1 Hz, 1H, *H*_ar_), 6.92–6.78 (m, 1H, *H*_ar_), 2.63 (s, 3H, PhC*H*_3_) ppm; ^13^C NMR (125 MHz, CDCl_3_) δ 160.0, 157.1, 155.0, 138.2, 137.4, 126.2, 121.2, 118.7, 118.6, 116.2, 23.7 ppm; IR (ATR) 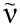: 3053, 2858, 2771, 2559, 2475, 2319, 1596, 1504, 1460, 1403, 1364, 1299, 1259, 1216, 1178, 1159, 1121, 1096, 1051, 989, 926, 883, 811, 746, 680 cm^−1^; UV (CH_2_Cl_2_) λ_max_, nm (log ε): 319 (2.84), 297 (2.80), 256 (2.92); HRMS–ESI (*m*/*z*): [M + H]^+^ calcd for C_12_H_11_NO, 186.0913; found, 186.0964; [M − H]^−^ calcd for C_12_H_10_NO, 184.0768; found, 184.0815.

**Methoxyphenylpyridine switch (10):** To a solution of dibromide **9** (20 mg, 0.022 mmol) and tetrakis(triphenylphosphine)palladium(0) (1 mg, 3.9 mol%) in dioxane (3 mL), a saturated potassium carbonate solution (0.2 mL) was added. The mixture was purged with argon for 10 min and afterwards 2-methoxyphenylboronic acid (14 mg, 0.092 mmol) was added and the mixture was heated to 80 °C for 4 h. After cooling to room temperature, water and ethyl acetate were added. The aqueous phase was extracted with ethyl acetate. The organic layers were combined and dried over magnesium sulfate, and the solvent was removed in vacuo. The residue was purified by column chromatography over silica gel (CH_2_Cl_2_/AcOEt/MeOH 75:25:2) and subsequently by HPLC. The product was obtained as a colorless solid (3.3 mg, 16%). ^1^H NMR (500 MHz, CDCl_3_) δ 8.54 (s, 1H, *H*_pyridine_), 8.14 (s, 1H, *H*_pyridine_), 7.38–6.93 (m, 8H, *H*_phenyl_, N*H*), 5.78 (s, 1H, *H*_pyridine_), 5.60 (s, 1H, *H*_pyridine_), 5.48–5.31 (m, 2H, imidazole-C*H_2_*-pyridine), 5.08–5.00 (m, 2H, imidazole-*CH*-NH), 4.92–4.89 (m, 2H, imidazole-C*H_2_*-pyridine), 4.60–4.52 (m, 2H, CO-C*H*-NH), 3.76–3.72 (m, 3H, OC*H****_3_***), 2.67–2.44 (m, 4H, C*H*(CH_3_)_2_, C*H_2_*C*H_2_*), 2.38–2.28 (m, 2H, C*H*(CH_3_)_2_), 1.70–1.61 (m, 2H, C*H_2_*C*H_2_*), 2.21–2.06 (m, 6H, imidazole-C*H****_3_***), 1.16–1.13 (m, 12H, CH(C*H_3_*)_2_), 1.00–0.92 (m, 12H, CH(C*H_3_*)_2_) ppm; ^13^C NMR (125 MHz, CDCl_3_) δ 171.11, 162.42, 162.25, 146.48, 146.34, 145.20, 144.90, 142.95, 134.57, 133.20, 131.62, 131.08, 130.53, 130.22, 111.00, 59.06, 55.54, 51.28, 51.10, 45.30, 44.64, 38.30, 34.05, 33.18, 30.92, 19.74, 19.70, 18.91, 18.81, 17.43, 13.34, 10.32, 10.11 ppm; IR (ATR) 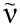: 3395, 2961, 2929, 2872, 2362, 2324, 1664, 1594, 1497, 1461, 1435, 1389, 1372, 1328, 1269, 1237, 1192, 1154, 1111, 1090, 1052, 1024, 957, 923, 892, 856, 797, 754, 730 cm^−1^; UV (CH_2_Cl_2_) λ_max_, nm (log ε): 295 (2.95); CD (CH_2_Cl_2_) λ nm (Δε M^−1^cm^−1^): 280 (+8.5), 252 (−26.3); HRMS–ESI (*m*/*z*): [M + H]^+^ calcd for C_49_H_61_^81^BrN_10_O_5,_ 951.4074; found, 951.4129; [M + Na]^+^ calcd for C_49_H_60_^81^BrN_10_O_5_Na, 973.3893; found, 973.3977.

**Hydroxyphenylpyridine switch (12):** To a solution of dibromide **9** (20 mg, 0.022 mmol) and tetrakis(triphenylphosphine)palladium(0) (1 mg, 3.9 mol %) in dioxane (3 mL), a saturated potassium carbonate solution (0.2 mL) was added. The mixture was purged with argon for 10 min and afterwards 2-hydroxyphenylboronic acid (14 mg, 0.102 mmol) was added and the mixture was heated to 80 °C for 9 h. After cooling to room temperature, water and ethyl acetate were added. The aqueous phase was extracted with ethyl acetate. The organic layers were combined and dried over magnesium sulfate, and the solvent was removed in vacuo. The residue was purified by column chromatography over silica gel (CH_2_Cl_2_/AcOEt/MeOH 75:25:2) and subsequently by HPLC. The product was obtained as a colorless solid (5.9 mg, 29%). ^1^H NMR (500 MHz, CDCl_3_) δ 8.48 (s, 1H, *H*_pyridine_), 8.22 (s, 1H, *H*_pyridine_), 7.45 (d, ^3^*J*_H,H_ = 7.3 Hz, 1H, *H*_phenyl_), 7.31–7.28 (m, 3H, *H*_phenyl_), N*H*-CH-imidazole), 7.13 (d, ^3^*J*_H,H_ = 7.3 Hz, 1H, *H*_phenyl_), 7.06 (d, ^3^*J*_H,H_ = 8.8 Hz, 2H, N*H*-CH-CO), 6.96–6.91 (m, 1H, *H*_phenyl_), 5.92 (s, 1H, *H*_pyridine_), 5.57 (s, 1H, *H*_pyridine_), 5.47 (d, 1H, ^2^*J*_H,H_ = 17.0 Hz, imidazole-C*H*_2_-pyridine), 5.36 (d, 1H, ^2^*J*_H,H_ = 17.7 Hz, imidazole-C*H*_2_-pyridine), 5.07 (d, 1H, ^2^*J*_H,H_ = 17.0 Hz, imidazole-C*H*_2_-pyridine), 4.98–4.88 (m, 3H, pyridine-C*H*-NH, imidazol-C*H_2_*-pyridine), 4.56–4.51 (m, 2H, CO-C*H*-NH), 3.19 (td, ^2^*J*_H,H_ = 14.2 Hz, ^3^*J*_H,H_ = 5.9 Hz, 1H, C*H*_2_CH_2_), 2.93 (td, ^2^*J*_H,H_ = 13.2 Hz, ^3^*J*_H,H_ = 5.5 Hz, 1H, CH_2_C*H*_2_), 2.56–2.43 (m, 2H, C*H*(CH_3_)_2_), 2.36–2.27 (m, 2H, C*H*(CH_3_)_2_), 2.24 (s, 3H, imidazole-C*H_3_*), 2.12 (s, 3H, imidazole-C*H_3_*), 1.99–1.91 (m, 1H, CH_2_C*H*_2_), 1.85 (td, ^2^*J*_H,H_ = 13.2 Hz, ^3^*J*_H,H_ = 4.7 Hz, 1H, C*H*_2_CH_2_), 1.15–1.14 (m, 12H, CH(C*H_3_*)_2_), 1.00–0.93 (m, 12H, CH(C*H****_3_***)_2_) ppm; ^13^C NMR (125 MHz, CDCl_3_) δ 171.05, 171.01, 162.29, 161.72, 156.33, 146.58, 146.28, 145.43, 143.18, 138.30, 135.53, 135.51, 135.03, 135.02, 133.42, 133.39, 133.13, 131.71, 130.92, 129.99, 129.89, 121.22, 119.52, 117.95, 59.06, 59.05, 58.99, 58.93, 51.21, 51.08, 44.75, 44.26, 37.57, 34.27, 33.23, 33.18, 30.81, 30.77, 19.72, 19.70, 18.91, 18.76, 18.69, 17.42, 17.41, 17.39, 10.19, 10.05 ppm; IR (ATR) 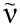: 3357, 3280, 2962, 2927, 2872, 2357, 2324, 1660, 1593, 1504, 1455, 1425, 1389, 1372, 1329, 1292, 1250, 1221, 1197, 1153, 1053, 1023, 991, 957, 891, 858, 835, 815, 800, 755, 732, 680 cm^−1^; UV (CH_2_Cl_2_) λ_max_, nm (log ε): 305 nm (2.73); CD (CH_2_Cl_2_) λ, nm (Δε M^−1^cm^−1^): 285 (+3.4), 268 (+6.7), 252 (−16.2); HRMS–ESI (*m*/*z*): [M + H]^+^ calcd for C_48_H_59_^81^BrN_10_O_5_, 937.3917; found, 937.3889; [M + Na]^+^ calcd for C_48_H_58_^81^BrN_10_O_5_, 959.3737; found, 959.3730.
